# Predictive Immunohistochemical Markers Related to Drug Selection for Patients Treated with Sunitinib or Sorafenib for Metastatic Renal Cell Cancer

**DOI:** 10.1038/srep30886

**Published:** 2016-08-04

**Authors:** Xin Ma, Lei Wang, Hongzhao Li, Yu Zhang, Yu Gao, Gang Guo, Kan Liu, Qingyu Meng, Chaofei Zhao, Dianjun Wang, Zhigang Song, Xu Zhang

**Affiliations:** 1 Department of Urology, State Key Laboratory of Kidney Diseases, Chinese PLA General Hospital/Chinese PLA Medical Academy, Beijing, P. R. China; 2 Department of Pathology, Chinese PLA General Hospital/Chinese PLA Medical Academy, Beijing, P. R. China

## Abstract

Targeted drug decisions in metastatic renal cell carcinoma are exclusively made on the basis of clinical criteria. We investigated whether these biomarkers (HIF-1α, HIF-2α, CAIX, VEGF, VEGFR1, VEGFR2, VEGFR3, PDGFB, PDGFRA, PDGFRB, CD31, CD44, bcl-xL, KIT, p21, CXCR4, PTEN, (CSF)-1R, RET, and FLT-3) can predictive the different effects between sunitinib and sorafenib treatments and are available to guide targeted drug selection. We enrolled all patients who underwent nephrectomy with postoperative sunitinib- or sorafenib-treatment at our institution from 2007 to 2012. Immunohistochemical approach was applied to assess the potential differential effects of immunostainings between sunitinib- and sorafenib-treated groups. We found that patients with high HIF-2α, CD31 expression showed greater relative PFS and OS benefit and patients with high CAIX expression presented greater relative OS benefit from sunitinib than from sorafenib, patients with high VEGFR1 or PDGFRB expression levels exhibited worse relative PFS benefit from sunitinib than from sorafenib. Namely high HIF-2α, CD31, and CAIX expression levels along with low VEGFR1 and PDGFRB expression levels improved the benefit of sunitinib treatment compared with sorafenib treatment. These results can identify whether patients can benefit more from sunitinib or sorafenib for drug selection guidance, eventually with precision medicine.

Metastatic renal cell carcinoma (mRCC) is a chemotherapy-resistant malignancy. Recent advances in molecular biology have led to the development of several novel agents to treat mRCC. As a consequence, monotherapy with interferon (IFN)-α or high-dose bolus interleukin (IL)-2 should no longer be routinely recommended as first-line therapy in mRCC, except in certain circumstances (e.g. lung metastasis, renal clear cell carcinoma (ccRCC), and long interval)^1^. To date, seven targeted drugs have been approved in the USA and Europe for treating mRCC: four vascular endothelial growth factor receptor (VEGFR)-targeted tyrosine kinase inhibitors (TKIs), including sorafenib, sunitinib, pazopanib, and axitinib; one anti-vascular endothelial growth factor (VEGF) monoclonal antibody, bevacizumab; and two mammalian target of rapamycin (mTOR) inhibitors, temsirolimus and everolimus^2^. Molecular-targeted therapy improved prognosis of mRCC compared with cytokine therapy. However, the length of response and survival benefit of targeted therapy varies considerably among patients. Patients may demonstrate different targeted-therapy sensitivities even with same pathological classification, clinical stage, dose, and mode of clinical treatment^3^,^4^.

VEGF signalling pathway inhibitors have been associated with various toxicities, including an increased risk of fatal adverse events^5^. With the realisation that limited criteria exist for prediction of response to a particular drug and that many sequential treatments are likely to be pursued^6^, the economic burden and adverse events of several drugs must be globally considered to achieve the optimum potential risk-to-benefit ratio for each patient. Clinical and translational studies to identify the phenotypic predictors of response to each drug are urgent. Partial treatment-resistant patients still respond to other targeted drugs in clinical practice. The differential effects of patients treated with differential targeted drugs for mRCC do exist. Identifying the biomarkers for efficacy is necessary to select suitable patients for this therapeutic approach.

A number of approaches such as blood-based biomarker, tissue-based biomarker, SNP biomarker, and cellular biomarkers are currently under investigation^2^. However, these markers typically only provide clinicians with risk assessment for a patient based on multiple criteria and are prognostic (i.e. providing information about independent outcome of treatment). A fraction of these are known to be predictive (i.e. providing information about efficacy of a specific treatment intervention). Nevertheless, the differential effects indicated by these predictive markers are typically compared with placebo or cytokine treatment but not with other targeted drugs. Hence, whether these biomarkers signify similar traits of distinguished therapeutic effects of other targeted drugs remains unclear. No guideline is available for drug selection. The hope and interest lie in the identification of accurate markers that can predict the responses to existing effective but toxic target therapies^7^,^8^,^9^,^10^.

Sunitinib and sorafenib were the first approved vascular endothelial growth factor-targeted drugs as first-line treatment of mRCC in China, with everolimus being the second-line drug. Sunitinib and sorafenib are all oral multikinase inhibitors with effects on tumour–cell proliferation and tumour angiogenesis. Sunitinib was identified as platelet-derived growth factor receptors (PDGFRA, PDGFRB), VEGFR1, VEGFR2, VEGFR3, stem cell factor receptor (KIT), Fms-like tyrosine kinase-3 (FLT-3), colony stimulating factor receptor Type 1 (CSF-1R), and the glial cell-line derived neurotrophic factor receptor (RET) inhibitor^9^. Sorafenib was initially identified as a Raf kinase inhibitor,10 which also inhibits VEGFR1, VEGFR2, VEGFR3, PDGFRB, Flt-3, RET, and KIT^11^. Sunitinib or sorafenib inhibition of these receptor tyrosine kinases has been demonstrated in biochemical and cellular assays, and inhibition of function has been demonstrated in cell proliferation assays.

We collected prognostic and predictive tissue-based biomarkers related to mRCC patients treated with sunitinib or sorafenib to validate whether these biomarkers can predictive the different effects between sunitinib and sorafenib treatments and are available to guide targeted drug selection. The following biomarkers were included in the study: hypoxia inducible factor 1, alpha subunit (HIF-1α), hypoxia inducible factor 2, alpha subunit (HIF-2α), carbonic anhydrase IX (CAIX), VEGF, VEGFR1, VEGFR2, VEGFR3, platelet-derived growth factor beta polypeptide (PDGFB), PDGFRA, PDGFRB, differentiated microvascular density (MVD, assessed by CD31 staining), CD44, BCL2-like 1 (bcl-xL), KIT, cyclin-dependent kinase inhibitor 1A (p21), chemokine (C-X-C motif) receptor 4 (CXCR4), and phosphatase and tensin homolog (PTEN). All these biomarkers have been established these prognostic or predictive function of RCC benefit from sunitinib or sorafenib in existing research^12^,^13^,^14^,^15^,^16^,^17^,^18^,^19^,^20^,^21^. For instance,’ C. D’Alterio found that high CXCR4 expression correlates with poor response to sunitinib in metastatic renal cancer^21^. A study of J. Garcia-Donas regarding the outcome of sunitinib treatment in advanced ccRCC found that PDGFRB was associated with better response evaluation criteria in solid tumours (RECIST) objective response to sunitinib^19^. In addition, we included FLT-3, CSF-1R, and RET in the present analysis because these biomarkers were targets of sunitinib or sorafenib^9^,^11^.

In this prospective analysis, we enrolled sunitinib- and sorafenib-treated groups. We aimed to use the IHC approach to identify predictive markers of drug selection in patients with mRCC (subgroups of patients receiving different degrees of relative benefit from sunitinib compared with sorafenib). This goal leads to selection of treatment with the optimum potential risk-to-benefit ratio for each patient.

## Patients and Methods

### Study design and patients

In this prospective cohort study, we consecutively enrolled adults (≥18 years) who underwent nephrectomy and had a pathologically confirmed diagnosis of renal clear cell carcinoma and distant organ metastasis. These patients were treated at the PLA General hospital from January 2007 to December 2012. Before the targeted treatment, all patients had a detailed history, physical examination, and laboratory parameters. Response to treatment was assessed by a treating doctor according to RECIST criteria trimestral^22^. Patients who were lost-to-follow-up are excluded from subsequent steps of analysis (three and 11 patients were excluded in the sunitinib and sorafenib groups, respectively). A total of 52 patients from the sunitinib-treated group and 55 from the sorafenib-treated group met the inclusion and exclusion criteria, had complete follow-up records, and were scheduled for therapy on a daily clinical practice setting. We aimed to use the IHC approach to identify predictive markers of drug selection in patients with mRCC (subgroups of patients receiving different degrees of relative benefit from sunitinib compared with sorafenib). This goal leads to selection of treatment with the optimum potential risk-to-benefit ratio for each patient. Written informed consent for a tumour-oriented study was obtained from all patients prior to sample collection. The study was approved by the Protection of Human Subjects Committee of Chinese People’s Liberation Army General Hospital, and the study was carried out in accordance with the Declaration of Helsinki.

### Tissue microarray and Immunohistochemistry

The formalin-fixed paraffin-embedded primary tumour specimens were obtained from the Department of Pathology at the PLA General Hospital. Three core-tissue biopsies with 1.0 mm in diameter were taken from the selected morphologically representative regions of each paraffin-embedded RCC and precisely arrayed using a custom-built instrument (Quick-Ray UT-06, UNITMA). Commercially available antibodies were used for all IHC studies. The following antibodies were studied: HIF-1α (1:200), HIF-2α (1:200), CAIX (1:1000), VEGF (1:100), VEGFR1 (1:200), VEGFR2 (1:300), VEGFR3 (1:200), PDGFB (1:200), PDGFRA (1:150), PDGFRB (1:300), CD31 (1:200), CD44 (1:200), bcl-xL (1:200), KIT (1:300), p21 (1:100), CXCR4 (1:900), PTEN (1:300), (CSF)-1R (1:25), RET (1:75), and FLT-3 (1:75). The stained TMA sections were analysed by two dedicated urologic pathologists (DJ Wang and ZG Song) while being unaware of the sample origin and clinical outcomes.

### IHC analysis

The immunostaining level was assessed by manual counting and was aided by analysis using Image-pro Plus 6.0 (IPP 6.0)^23^. The accuracy of this software has not only been verified by some authors but has also been recently applied to numerous aspects in biomedicine. The measurement parameter was integrated optical density (IOD). The function of irregular automated optical inspection (irregular AOI) was applied using the IPP 6.0 software to score and rule out non-target staining. Interesting targets included the tumour cell and adjacent fibroblasts. All images analysed using IPP 6.0 were verified by pathologists. Bcl-xL, which mainly stains the cell nuclei, was determined by counting 1,000 cells in 10 large graticules visible in the microscope. The results were semiquantitatively reported on a scale of 0–3 for intensity, where 0 was negative, 1 was weak, 2 was moderate, and 3 was strong. The percentage of tumour staining was reported as 0–100% in increments of 10%. A composite score was formed using the product of the intensity and percentage of tumour staining. Differentiated MVD was determined by IHC staining of CD31, and MVD was derived by counting each vessel identified within the selected areas, including any stained endothelial cell or endothelial-cell cluster that was separated from adjacent microvessels. Vessel lumens were not required for identifying a structure as a microvessel. Microvessels in necrotic or sclerotic areas within a tumour were not considered in vessel evaluations. Measurements for three cores per sample were averaged for the analysis. The mean value of the IOD or vessel counts in the selected ‘hotspots’ was retained as the final value.

### Statistical analysis

We defined progression free survival (PFS) as the time between the first day of treatment and the date of radiological progressive disease (PD), clear clinical evidence of PD, or death. Patients who had not progressed at database closure were censored during the final follow-up. If the PD date was unknown, we censored PFS at the last tumour assessment. Overall survival (OS) was defined as the time between the first day of system treatment and the date of death.

An ideal marker can reliably separate patients, and its expression should provide enough difference. Therefore, we created a scatter chart with IOD values or composite scores of all IHC markers (MVD was not included) to observe the distribution of IHC marker expression. Those with less coefficient of variation (CV) were excluded.

**Figure 1:**
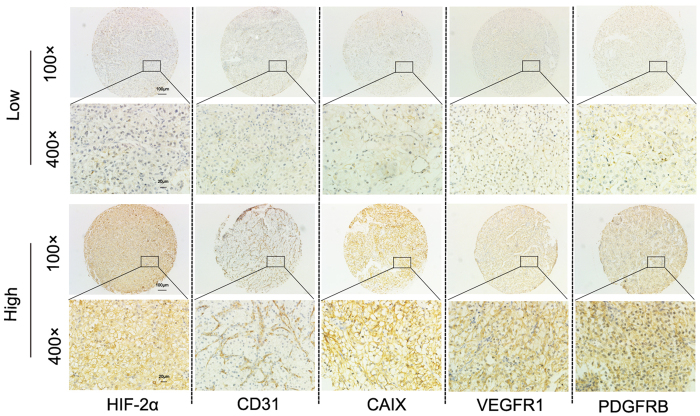
High expression and low expression of HIF-2α, CD31, CAIX, VEGFR1, and PDGFRB in metastatic renal-cell carcinoma. Representative immunohistochemistry of tissue core array stained with HIF-2α, CD31, CAIX, VEGFR1, and PDGFRB antibody.

Cut-off estimation aims to assess potential differential effects of individual immunostainings among different treatment groups. The same standard was needed to define high- and low-expression subgroups in different treatment groups. (Fig. 1) Therefore, individual IHC markers were analysed on the basis of the results of median cut-off among all 107 patients.

Ultimately, candidate predictive markers related to targeted drug selection were assessed. To establish any IHC marker as a prognostic marker for sunitinib- and/or sorafenib-treated groups, We used the Kaplan–Meier method to analyse PFS and OS, and the Cox regression model to verify significant differences noted in the Kaplan–Meier curves for sunitinib- and sorafenib-treated groups between high- and low-expression subgroups defined by the respective median IOD value. We used multivariable analysis by including age, sex, BMI, T stage, Fuhrman grade, and Memorial Sloan–Kettering Cancer Center (MSKCC) score as covariates (clinical factors that were associated with p < 0·05 with a specific variable were used as covariates for that specific variable). To establish any IHC marker as a predictive marker, we included a treatment versus immunostaining interaction term in the Cox model analysis for both PFS and OS to assess the potential differential effects of immunostainings between the two treatment groups, with treatment group and immunostainings as two other independent variables. IHC markers with a significant p_interaction_ value with treatment were regarded as predictor of drug selection. A two-sided p value of less than 0.05 was regarded as significant in all stages of this analysis. All the statistical analyses (apart from clustering) were conducted using SPSS version 19.0 and GraphPad 6.0.

## Results

A total of 107 patients were enrolled in this study between January 2007 and December 2012, and the follow-up database was closed in July 2015. After a median follow-up of 47 months (range of 29–102 months, IQR33–54 months), the median PFS and OS of the 107 patients was 13.6 months (1.5– >102 months, 6.7– >35 months) and 30.5 months (1.5– >102 months, 18– >41 months), respectively. Common metastatic sites, risk factors, Fuhrman grades, and T stage between the sunitinib and sorafenib groups were approximate. Table 1 shows the clinical characteristics of the enrolled patients in the three groups.

**Figure 2:**
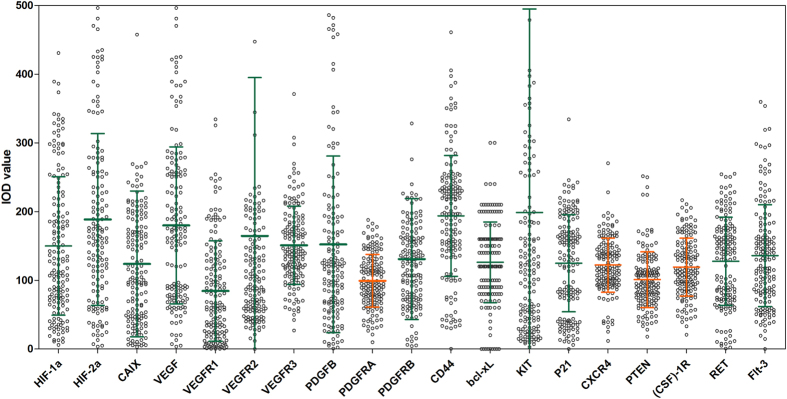
Scatter chart with IOD values or composite scores of 19 IHC markers. Line at Mean with Standard Deviation (SD), Coefficient of Variation(SD/Mean) as follows: CV(HIF-1α) = 0.67, CV(HIF-2α) = 0.66; CV(CAIX) = 0.86; CV(VEGF) = 0.64; CV(VEGFR1) = 0.86; CV(VEGFR2) = 1.40; CV(VEGFR3) = 0.48; CV(PDGFB) = 0.84; CV(PDGFRA) = 0.39; CV(PDGFRB) = 0.67; CV(CD44) = 0.45; CV(bcl-xL) = 0.46; CV(KIT) = 1.49; CV(P21) = 0.57; CV(CXCR4) = 0.32; CV(PTEN) = 0.32; CV((CSF)-1R) = 0.35; CV(RET) = 0.50; CV(Flt-3) = 0.55.

The scatter chart shows the expression distribution of 19 markers (Fig. 2). The expression levels of PDGFRA (CV = 0.39), PTEN (CV = 0.32), (CSF)-1R (CV = 0.35), and CXCR4 (CV = 0.32) in a fitted normal distribution, and this coefficient of variation is small, thus were excluded from the analysis.

**Figure 3:**
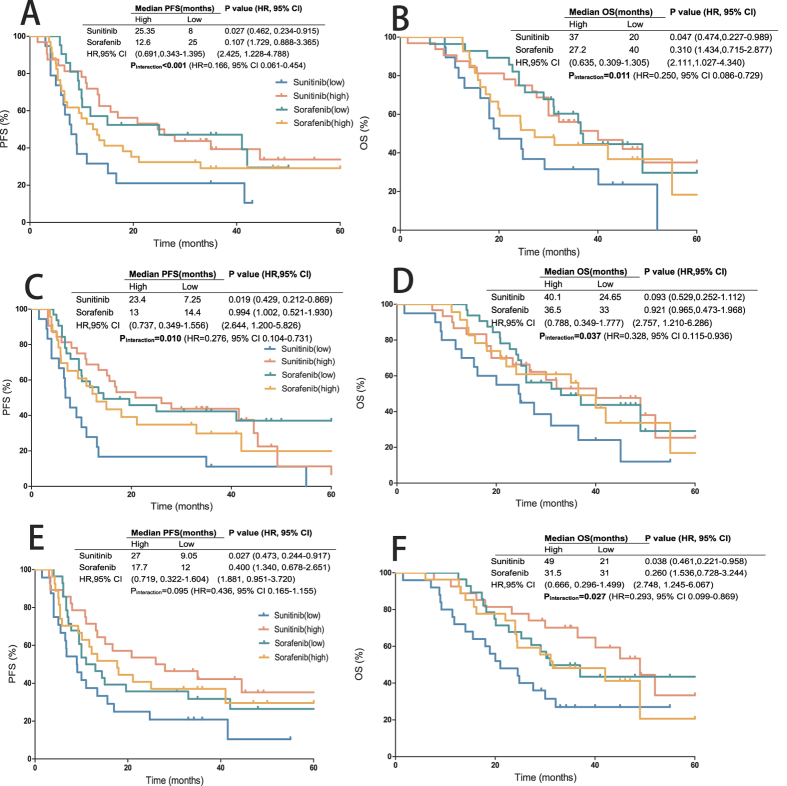
HIF-2α, CD31 and CAIX associated with PFS and OS. (**A**) HIF-2α with PFS; (**B**) HIF-2α with OS; (**C**) CD31 with PFS; (**D**) CD31 with OS; (**E**) CAIX with PFS; (**F**) CAIX with OS. HRs and p values are both come from Cox model analysis.

**Figure 4:**
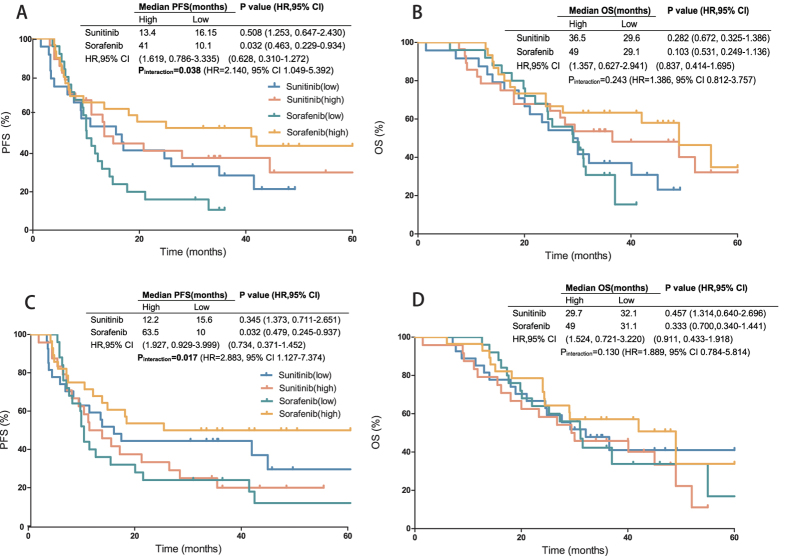
VEGFR1 and PDGFRB associated with PFS and OS. (**A**) VEGFR1 with PFS; (**B**) VEGFR1 with OS; (**C**) PDGFRB with PFS; (**D**) PDGFRB with OS. HRs and p values are both come from Cox model analysis.

Tables 2 and 3 show the 16 IHC markers staining as a dichotomous variable correlated with PFS and OS. Multivariable analysis showed the prognostic function of each IHC marker for sunitinib- and sorafenib-treated groups (Tables 2 and 3). Then we compared the sunitinib group with the sorafenib group to identify the predictive markers related to targeted drug selection. HIF-2α, CD31 were the IHC markers with significant predictive value for both PFS and OS benefits. CAIX (p_interaction_ = 0.027) exhibited a significant predictive value for OS benefit, whereas VEGFR1 (p_interaction_ = 0.038) and PDGFRB (p_interaction_ = 0.017) showed significant predictive value for PFS benefit. In the sunitinib group, patients with high HIF-2α expression displayed a longer PFS than those with low HIF-2α expression (25.35 months vs 8 months; p = 0.027). An inverse effect was noted in the sorafenib group (12.6 vs 25 months; p = 0.107). Patients with high HIF-2α expression showed a greater relative PFS benefit from sunitinib than from sorafenib (hazard ratio: 0.691, 95% CI: 0.343–1.395). On the contrary, patients with low HIF-2α expression presented a worse relative PFS benefit from sunitinib than from sorafenib (2.425, 1.228–4.788). Equivalent difference was also noted in the OS benefit (p_interaction_ = 0.011). Patients with high HIF-2α expression displayed a longer OS than those with low HIF-2α expression in the sunitinib group (37 months vs 20 months; p = 0.047). And inverse effect was noted in the sorafenib group (27.2 vs 40 months; p = 0.310) (Fig. 3A,B). Approximate predictive value was also noted in CD31 for both PFS and OS benefits. Patients with high CD31 expression showed a greater relative PFS and OS benefits from sunitinib than from sorafenib, whereas those with low CD31 expression presented a worse relative PFS and OS benefits (hazard ratio and 95% CI on Fig. 3C,D and Tables 2 and 3). CAIX exhibited a significant predictive value for the OS benefit (p_interaction_ = 0.027) but did not attain enough significance in the PFS benefit (p_interaction_ = 0.095). Patients with high CAIX expression showed a greater relative OS benefit from sunitinib than from sorafenib (0.666, 0.296–1.499). Low concentrations of CAIX expression presented a worse relative OS as well (2.748, 1.245–6.067) (Fig. 3E,F). Inverse difference was noted for VEGFR1 (p_interaction_ = 0.038) and PDGFRB (p_interaction_ = 0.017) in PFS benefit. In the sorafenib group, patients with high VEGFR1 expression showed a much longer PFS than those with low VEGFR1 expression (41 months vs. 10.1 months; p = 0.032), whereas this effect was not noted in the sunitinib group (13.4 vs. 16.15 months; p = 0.508) (Fig. 4A,B). Equivalent predictive value was also noted in PDGFRB for the PFS benefit (hazard ratio and 95% CI on Fig. 4C,D and Tables 2 and 3). To compare the power of interaction action of each immunostainings, we analysed the HR values of each interaction term. HIF-2α showed the most notable HR value of interaction action with treatment for both PFS and OS benefits, and the hazard ratios of drug comparison in the lower HIF-2α group and higher HIF-2α group are in different direction.

## Discussion

In clinical practice, the patient’s genetic information used to lead the treatment decisions of personalized treatment increasingly. Oncology transited to accurate drug phase rapidly. Nowadays researchers recognize that tiny genotype alteration may cause distinguished drug responses for different cancers. A number of studies reported that how gene researches apply to personalized treatment recently. E.g. 2014 National Comprehensive Cancer Network (NCCN) Guideline Insights highlighted the important role of KIT or PDGFRA mutation status in treatment decisions of gastrointestinal stromal tumours with imatinib and/or sunitinib^24^; The antibody-drug conjugate trastuzumab emtansine has improved outcomes in patients with human epidermal growth factor receptor 2-positive metastatic breast cancer compared with trastuzumab-based therapy, as demonstrated in phase III studies^25^; BRAF-positive non-small cell lung cancer (NSCLC) is sensitive to Dabrafenib. Anaplastic lymphoma kinase (ALK)-positive NSCLC is sensitive to treatment with an ALK-tyrosine kinase inhibitor^26^.

However, treatment decisions in mRCC are exclusively made on the basis of clinical criteria. Emergence of new targeted drugs options for mRCC has increased the need to prospectively identify populations of patients that are likely to benefit from the specific target treatment and predictive markers related to targeted drug selection. An ideal predictive biomarker is the one that is easily and unambiguously measured and reliably separates patients who will benefit from a specific approach to those who will benefit from an alternative approach^27^. IHC is a simple, inexpensive, and reliable assay. Given that renal cell tumours are usually treated by partial or radical nephrectomy, tumour tissue is routinely available for IHC. Sunitinib-treated patients showed longer PFS and OS than sorafenib-treated patients in multiple phases 2 and 3 trials^9^,^10^. In the current study, the sunitinib-treated group displayed similar median PFS and OS compared with the sorafenib-treated group. This finding may be ascribed to the exclusion of patients who were lost-to-follow-up (three in sunitinib group and 11 in sorafenib group). Those patients who were lost-to-follow-up usually demonstrate poor prognosis. The sunitinib- or sorafenib-treated group having slightly longer median PFS and OS than in phase 3 trial may come from two aspects. One is exclusion of those lost-to-follow-up patients; The other is that all the included patients underwent nephrectomy or cytoreductive nephrectomy (CN). In a meta-analysis of two randomised controlled trials comparing CN plus systemic treatment versus systemic treatment alone, a significant increase was observed in long-term survival of patients treated with CN^28^.

Five IHC markers (HIF-2α, CD31, CAIX, VEGFR1, and PDGFRB) were identified as predictive markers related to targeted drug selection. High HIF-2α, CD31, and CAIX expression levels along with low VEGFR1 and PDGFRB expression levels improved the benefit of sunitinib treatment compared with sorafenib treatment. On the contrary, low HIF-2α, CD31, and CAIX expression levels along with high VEGFR1 and PDGFRB expression predicted improved benefit relative to sorafenib. High RET and low bcl-xL expression demonstrated the predictive trend of improved benefit relative to sunitinib compared with sorafenib, but p_interaction_ was not significant. High levels of HIF-2α confer more favourable response to sunitinib therapy in two articles and agreed well with the present results^13^,^14^. We further discovered that this effect was not observed in the sorafenib therapy. But the inverse tendency was noted, patients with high HIF-2α expression displayed a shorter PFS and OS than those with low HIF-2α expression in the sorafenib group. High CAIX expression is associated with a better prognosis in localised RCC and mRCC^12^, similar to the tendency reflected in our trail, while high CAIX expression predicted more benefit relative to sunitinib treatment than sorafenib treatment. CD31 was associated with better prognosis in RCC in multiple studies^15^, we further found the differential effects of CD31 between sunitinib and sorafenib treatments. CD31 was associated with better sunitinib treatment than sorafenib treatment. The study by J. Garcia-Donas on the outcome of sunitinib treatment in advanced ccRCC found that PDGFRB was associated with better sunitinib RECIST objective response^19^. In the present study, this effect was not noted in the sunitinib group but was noted in the sorafenib group. This is possibly because of different evaluation methods. J. Garcia-Donas used objective response as effect indicator; that effect was not noted in PFS and OS. Moreover, the study of J. Garcia-Donas did not find VEGFR1 related to prognosis in sunitinib treatment. The present study reached the same conclusion, and found that VEGFR1 was associated with better sorafenib treatment effect furthermore.

**Figure 5:**
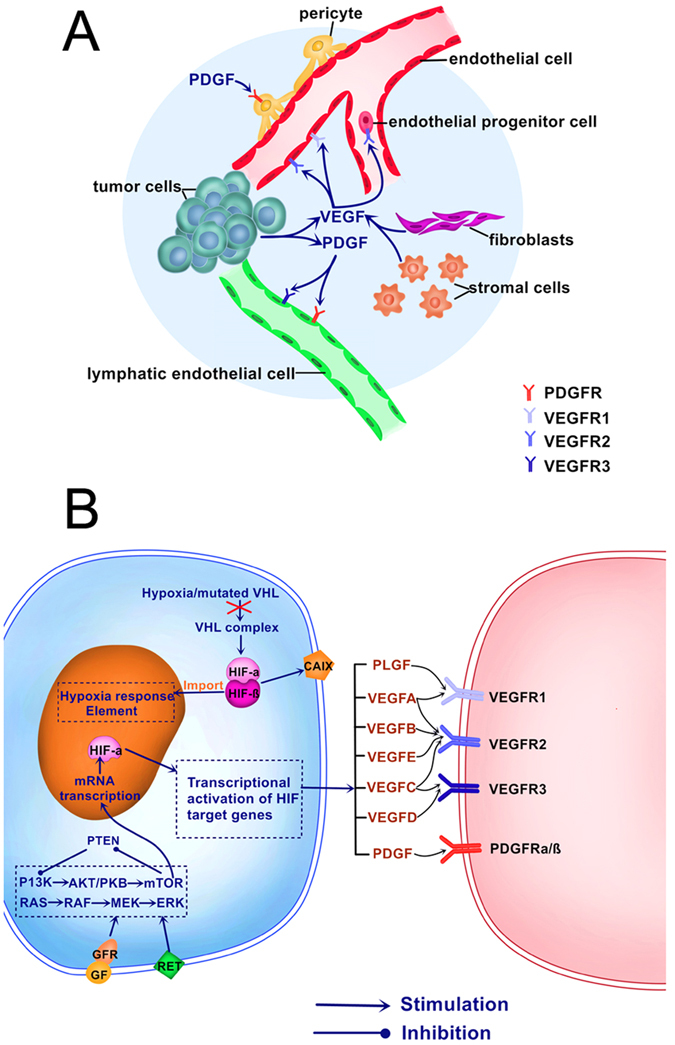
Biologic pathways in renal cell carcinoma and tyrosine kinase receptors targeted by VEGF signalling pathway inhibitors. (**A**) Tyrosine kinase receptors involved in angiogenesis and lymphangiogenesis targeted by VEGF signalling pathway inhibitors. Multiple cellular subtypes, including endothelial cells, pericytes, tumour cells, fibroblasts and endothelial progenitor cells, are implicated in tumour angiogenesis. Signalling through vascular endothelial growth factor receptors and platelet-derived growth factor receptors leads to endothelial cell growth, migration and survival. Tumour lymphangiogenesis is mainly driven through VEGFC/VEGFR3 and PDGF/PDGFR signalling in lymphatic endothelial cells. (**B**) Biologic pathways and markers in renal cell carcinoma: AKT/PKB = akt/protein kinase B (gene); ERK = extracellular signal-regulated kinase; GF = growth factor; GFR = growth factor receptor; MEK = methyl ethyl ketone.

The above five proteins have been identified as key factors of hypoxia and angiogenesis. They work cooperatively or function independently in different circumstance. As shown in Fig. 5, In clear cell RCC, the upregulation of VEGF mRNA levels is expected due to HIF-1α dysregulation as a result of VHL protein loss in addition to the hypoxic environment^3^. HIF-2α is one of the most investigated member of the HIF-α subunits, ccRCC has an inactivated VHL gene express either in the HIF-2α alone or in both HIF-1α and HIF-2α. VEGF–VEGFR signalling plays an important role in angiogenesis and vasculogenesis, VEGFR1 is expressed on tumour cells and binds to VEGFR-A, VEGFR-B, and placental growth factor. And that PDGFRB have been suggested to play crucial roles in tumour–vessel stability by recruiting pericytes to newly formed vessels^29^. CAIX is a HIF-1α-regulated transmembrane protein^12^. MVD gives important information on tumour vascularisation, which might be important for response to TKI treatment.

The mechanisms by which HIF-2α, CD31, CAIX, VEGFR1, and PDGFRB may predict drug-specific benefit remains unclear. The cause may be the different inhibiting effects to several kinases between sunitinib and sorafenib. *In vitro* studies found that differential regulation of sunitinib or sorafenib targets predicts its tumour-type-specific effect on endothelial and/or tumour cell apoptosis, and showed that molecular targets could be used as biomarkers capable of assessment of therapeutic response^30^. We need to dig deeper to find out these mechanisms. Molecular events that can unveil the biologic heterogeneity underlying the varied clinical behaviour of RCC may help improve individualised prognostication and risk-stratified clinical decision-making.

Protein translation is a biological process occurring in the cytoplasm and a transcription process occurring in the nucleus. Therefore, the absolute expression and sub-cellular localisation staining of IHC markers may predict clinicopathological features and outcome in ccRCC. We did not analyse sub-cellular localisation staining of IHC markers, but this method should be involved in future research. Comparative study between other targeted drugs such as pazopanib, bevacizumab, and temsirolimus should be conducted as well, and others kinds of markers (e.g. blood-based biomarker, cellular biomarkers, even circulating tumour DNA) should be involved in future research.

Finally, this study did not include an external validation. Small sample sizes, long time spans, and single-centred study decreased the power. Because the patients were all from the yellow race, the relevance of these predictive roles needs to be assessed in other ethnic groups. However, we only included patients with clear-cell cancer component tumours to ensure homogeneity of our data and excluded patients who were lost-to-follow-up. Response to treatment was assessed by a treating doctor. These factors are probably the major contributors to the robustness of the results, with statistically significant outcomes that persisted after adjustment for multiple testing.

## Conclusion

High HIF-2α, CD31, and CAIX expression levels along with low VEGFR1 and PDGFRB expression levels improved the benefit of sunitinib treatment compared with sorafenib treatment. On the contrary, low HIF-2α, CD31, and CAIX expression levels along with high VEGFR1 and PDGFRB expression levels improved the benefit of sorafenib treatment compared with sunitinib treatment. If confirmed, these results can identify drug resistant patients early, and identify whether a patient may benefit from a specific targeted therapy and serve as a guide for drug selection, eventually with precision medicine.

### Take home message

We enrolled sunitinib-treated and sorafenib-treated groups meanwhile, to identify predictive markers of drug selection in patients with mRCC (subgroups of patients receiving different degrees of relative benefit from sunitinib compared with sorafenib).

## Additional Information

**How to cite this article**: Ma, X. *et al*. Predictive Immunohistochemical Markers Related to Drug Selection for Patients Treated with Sunitinib or Sorafenib for Metastatic Renal Cell Cancer. *Sci. Rep.* **6**, 30886; doi: 10.1038/srep30886 (2016).

## Figures and Tables

**Table 1:** Clinical characteristics of patients.

	Sunitinib (N = 52)	Sorafenib (N = 55)
Median age (years)	51(18–81,44–62)	58(29–79,47–64)
Sex (male)	40(78%)	41(75%)
Median follow-up (months)	49(32–78, 33–59)	46(29–102, 34–50)
Median PFS	14.25(1.5– >78, 6.6– >35)	13.4(3.7– >102, 6.7– >36)
Median OS	30(1.5– >78, 17.1– >41.6)	31.5(3.7– >102, 19.4– >41)
Common metastasis sites		
Lung	45(86%)	43(78%)
Lymph nodes	25(48%)	23(42%)
Bone	11(21%)	13(24%)
Liver	9(17%)	6(11%)
Risk factors*		
0 (favourable)	10(19%)	12(22%)
1–2 (intermediate)	38(73%)	39(71%)
≥3 (poor)	4(8%)	4(7%)
Fuhrman grade		
1	1(2%)	4(7%)
2	28(54%)	27(49%)
3	19(36%)	19(35%)
4	4(8%)	5(9%)
T stage		
1–2	28(54%)	31(56%)
≥3	24(46%)	24(44%)

Data are median (range, IQR) or n (%). *Risk groups according to Memorial Sloan-Kettering Cancer Center prognostic factors, T stage is pathological stage.

**Table 2:** 16 IHC markers and PFS in the biomarker population.

		PFS (months)		P value (*HR, 95% CI*)		P_interaction_
		Sunitinib	Sorafenib	Sunitinib	Sorafenib	
HIF-1α	High	11	13	0.239 (*1.496, 0.765–2.928*)	0.994 (*1.002, 0.521–1.930*)	0.847
	Low	18.9	14.4			
HIF-2α	High	25.35	12.6	0.027 (*0.462, 0.234–0.915*)	0.107 (*1.729, 0.888–3.365*)	<0.001
			Low	8	25	
CAIX	High	27	17.7	0.027 (*0.473, 0.244–0.917*)	0.400 (*1.340, 0.678–2.651*)	0.095
			Low	9.05	12	
VEGF	Low	13.4	10	0.218 (*0.659, 0.340–1.280*)	0.027 (*0.423, 0.197–0.909*)	0.347
			High	14.9	undefined	
VEGFR1	High	13.4	41	0.508 (*1.253, 0.647–2.430*)	0.032 (*0.463, 0.229–0.934*)	0.038
			Low	16.15	10.1	
VEGFR2	High	15.6	13.4	0.908 (*0.958, 0.465–1.976*)	0.638 (*1.197, 0.567–2.528*)	0.187
			Low	11	13.3	
VEGFR3	High	11	9.75	0.303 (*1.434, 0.722–2.845*)	0.325 (*1.391, 0.721–2.683*)	0.263
			Low	18.9	21.1	
PDGFB	High	17	15	0.599 (*0.776, 0.259–1.930*)	0.384 (*1.393, 0.660–2.939*)	0.087
			Low	11	29.5	
PDGFRB	High	12.2	63.5	0.345 (*1.373, 0.711–2.651*)	0.032 (*0.479, 0.245–0.937*)	0.017
			Low	15.6	10	
CD31	High	23.4	13	0.019 (*0.429, 0.212–0.869*)	0.994 (*1.002, 0.521–1.930*)	0.010
			Low	7.25	14.4	
CD44	High	10.9	11	0.026 (*2.259, 1.101–4.637*)	0.067 (*1.949, 0.955–3.975*)	0.838
			Low	41.5	25	
bcl-xL	High	6.8	14.4	0.001 (*3.530, 1.739–7.168*)	0.868 (*1.057, 0.551–2.028*)	0.084
			Low	44.5	12.35	
KIT	High	13.4	9.4	0.8678 (*0.867, 0.478–1.546*)	0.198 (*1.558, 0.793–3.063*)	0.622
			Low	16.7	18	
p21	High	11	9.3	0.039 (*2.120, 1.037–4.333*)	0.135 (*1.674, 0.851–3.291*)	0.411
			Low	45.3	41	
RET	High	12.05	9.5	0.377 (*1.389, 0.670–2.876*)	0.065 (*2.051, 0.955–4.405*)	0.090
			Low	16.7	undefined	
FLT-3	High	14.25	14.85	0.747 (*0.895, 0.456–1.756*)	0.921 (*1.034, 0.538–1.986*)	0.422
			Low	14.5	13.4	

Survival data are median, p values and HRs for sunitinib- and sorafenib-treated groups are both come from multivariable analysis, they compare higher biomarker group to lower biomarker group; p_interaction_ values are come from multivariate analysis (Cox model analysis) to assess the potential differential effects of immunostainings between the two treatment groups.

**Table 3:** 16 IHC markers and OS in the biomarker population.

		OS (months)		P value		P_interaction_
		Sunitinib	Sorafenib	Sunitinib	Sorafenib	
HIF-1α	High	29.2	32.5	0.849 (*1.073, 0.521–2.209*)	0.921 (*0.965. 0.473–1.968*)	0.737
			Low	36.5	38.5	
HIF-2α	High	37	27.2	0.047 (*0.474, 0.227–0.989*)	0.310 (*1.434, 0.715–2.877*)	0.011
			Low	20	40	
CAIX	High	49	31.5	0.038 (*0.461, 0.221–0.958*)	0.260 (*1.536, 0.728–3.244*)	0.027
			Low	21	31	
VEGF	High	36.5	55	0.248 (*0.652, 0.315–1.348*)	0.024 (*0.368, 0.154–0.879*)	0.215
			Low	32.1	25.8	
VEGFR1	High	36.5	49	0.282 (*0.672, 0.325–1.386*)	0.103 (*0.531, 0.249–1.136*)	0.243
			Low	29.6	29.1	
VEGFR2	High	36.5	42	0.897 (*0.950, 0.435,2.073*)	0.515 (*1.308, 0.582–2.940*)	0.138
			Low	24.8	31.5	
VEGFR3	High	29.4	28.15	0.989 (*1.005, 0.478–2.114*)	0.765 (*1.119, 0.535–2.341*)	0.127
			Low	33.25	37	
PDGFB	High	36.5	37	0.373 (*0.829, 0.352–1.912*)	0.490 (*1.343, 0.581–3.105*)	0.121
			Low	27.6	35	
PDGFRB	High	29.7	49	0.457 (*1.314, 0.640–2.696*)	0.333 (*0.700, 0.340–1.441*)	0.130
			Low	32.1	31.1	
CD31	High	40.1	36.5	0.093 (*0.529, 0.252–1.112*)	0.921 (*0.965, 0.473–1.968*)	0.037
			Low	24.65	33	
CD44	High	24.8	36	0.033 (*2.380, 1.071–5.289*)	0.476 (*1.302, 0.630–2.691*)	0.562
			Low	52	49	
bcl-xL	High	21	36.5	0.011 (*2.626, 1.244–5.543*)	0.477 (*0.772, 0.377–1.577*)	0.465
			Low	49	36	
KIT	High	49	19.15	0.7140 (*0.835, 0.324–1.334*)	0.508 (*1.280, 0.616–2.660*)	0.622
			Low	28.4	37	
p21	High	29.2	31	0.185 (*1.690, 0.778–3.671*)	0.276 (*1.494, 0.726–3.075*)	0.600
			Low	40.1	46	
RET	High	28.05	25.2	0.236 (*1.610, 0.732–3.540*)	0.039 (*2.377, 1.044–5.411*)	0.452
			Low	32.1	49	
FLT-3	High	36.5	42	0.867 (*0.939, 0.447–1.970*)	0.600 (*0.828, 0.410–1.676*)	0.564
			Low	29.4	30.6	

Survival data are median, p values and HRs for sunitinib- and sorafenib-treated groups are both come from multivariable analysis, they compare higher biomarker group to lower biomarker group; p_interaction_ values are come from multivariate analysis (Cox model analysis) to assess the potential differential effects of immunostainings between the two treatment groups.
